# A Hierarchical Framework Approach for Voice Activity Detection and Speech Enhancement

**DOI:** 10.1155/2014/723643

**Published:** 2014-05-12

**Authors:** Yan Zhang, Zhen-min Tang, Yan-ping Li, Yang Luo

**Affiliations:** ^1^College of Computer Science and Technology, Nanjing University of Science and Technology (NUST), Nanjing 210094, China; ^2^College of Information Technology, Jinling Institute of Technology (JIT), Nanjing 211169, China; ^3^College of Telecommunications and Information Engineering, Nanjing University of Posts and Telecommunications (NUPT), Nanjing 210046, China

## Abstract

Accurate and effective voice activity detection (VAD) is a fundamental step for robust speech or speaker recognition. In this study, we proposed a hierarchical framework approach for VAD and speech enhancement. The modified Wiener filter (MWF) approach is utilized for noise reduction in the speech enhancement block. For the feature selection and voting block, several discriminating features were employed in a voting paradigm for the consideration of reliability and discriminative power. Effectiveness of the proposed approach is compared and evaluated to other VAD techniques by using two well-known databases, namely, TIMIT database and NOISEX-92 database. Experimental results show that the proposed method performs well under a variety of noisy conditions.

## 1. Introduction

Voice activity detection (VAD), which is a strategy to automatically identify the presence of speech frames in a given signal, is a fundamental step in speech processing applications. The problem of VAD has been widely studied since 1970s because of its consequence in many different applications, such as speech query and speaker recognition. The main objective of a VAD system is to correctly separate speech and nonspeech patterns in a given signal. Accurate VAD can compensate for the harmful effect and improve the performance of speech recognition in real or noisy environment.

In the past decade, plentiful VAD techniques have been developed to achieve better performance in real or noisy environments. In addition, some surveys of the existing algorithms are also available in this domain. Sahidullah and Saha [1] systematically reviewed some popular VAD techniques and their applications on NIST speech corpora in their work. Han et al. [2] investigated the effect of VAD on noise reduction performance by applying current VAD algorithms that have shown remarkable performance in single microphone noise reduction.

Generally, VAD scheme could be divided into three broad categories, namely, time-domain, frequency-domain, and statistical approaches. Many of the time-domain and frequency-domain approaches are based on heuristic rules which reflect the production characteristics on several parameters, such as linear predictive coding (LPC), short-time energy, zero crossing rate (ZCR), Mel-frequency cepstral coefficients (MFCC), spectral entropy, and periodicity measures. Energy-based VAD techniques are very straightforward, and they are widely used in speech and speaker recognition applications. Hsieh et al. [3] proposed an energy-based VAD algorithm with the GMSS (grey magnitude spectral subtraction). Simulations results show that the proposed approach is able to work properly in low signal-to-ratio (SNR) cases where the white noise is considered. Shen et al. [4] first used an entropy-based parameter for voice activity detection under low SNR conditions. Their experimental results revealed that the spectral entropy of a speech signal differs significantly from that of a nonspeech signal. Cho and Kim [5] proposed an enhanced voice activity detection scheme by using some perceptual features and MFCC. Performances were compared with state-of-the-art techniques in real-world environment. Ishizuka et al. [6] proposed a noise robust voice activity detection technique called PARADE (PAR based Activity DEtection) that employs the periodic component to aperiodic component ratio (PAR). Comparisons of the performance for noisy speech show that the proposed approach achieved significantly higher word accuracies than those achieved by MFCC based feature extraction methods. Sadjadi and Hansen [7] proposed a robust and unsupervised VAD solution that leverages four different perceptual spectral flux features. Experimental results indicate that the proposed scheme provides superior and consistent performance across various noise types and distortion levels. The harmonic structure-related information is well-known bearers of acoustic information for increasing the noise robustness. Fukuda et al. [8] proposed a new approach including both the long-term temporal and the static harmonic structure features with statistical models. The proposed approach led to considerable improvements under low SNR conditions as compared with the other state-of-the-art VAD approaches.

The statistical model based VAD approaches, such as hidden Markov models (HMM), Gaussian mixture models (GMM), support vector machine (SVM), and likelihood ratio test (LRT), are considered as attractive approaches for noisy speech. These methods do not depend critically on threshold setting but require training data for different types of background noises. Performance of these approaches depends on the choice of the probability distributions and the ability to estimate the parameters of the noise distribution [9]. Kim and Chang [10] proposed a statistical VAD method in a high-dimensional kernel feature space by a nonlinear mapping. A Gaussian density model is presented using kernel principal component analysis to represent the nonlinear characteristics of the speech signal. Górriz et al. [11] presented a novel VAD based on a multivariate complex Gaussian (MCG) observation model and defined an optimal likelihood ratio test (LRT) involving multiple and correlated observations. Wu and Zhang [12] proposed a multiple kernel support vector machine (MK-SVM) method for multiple feature based VAD. Bao and Zhu [13] combined harmonic structure information and the high order statistics (HOS) with Gaussian mixture model based hidden Markov model (HMM/GMM) for efficient speech/nonspeech classification.

Since the performance of features may be different in the decision process under different conditions, some researches proposed feature voting schemes to improve the degree of reliability and discriminative power. Moattar and Homayounpour [14] employed several short-term speech/nonspeech discriminating features in a weighted voting paradigm to achieve a reliable performance in different environments. Haghani and Ahadi [15] proposed a feature fusion approach for noise robust voice activity detection. Experimental evaluation confirmed the potential performance of the proposed approach under noisy conditions.

In this study, a hierarchical framework approach for VAD and speech enhancement is proposed. The proposed system was composed of three blocks. In the speech enhancement block, the modified Wiener filter (MWF) approach is utilized for noise reduction. A priori SNR is estimated by the decision directed (DD) method. For the feature extraction and voting block, several discriminating short-term features were extracted in various noisy conditions. Since some features are more appropriate for a specific noisy condition, these features were then employed in a voting paradigm for the consideration of reliability and discriminative power. The “one-against-one” approach [5] of support vector machine (SVM) was utilized for separating speech/nonspeech segments in the classification block. The paper is organized as follows.  Section 2 provides a brief introduction of the modified Wiener filter approach and the decision directed method for speech enhancement.  Section 3 introduces the feature extraction techniques to be used in the paper, the energy-based VAD techniques, spectral entropy, and harmonic structure information. In Section 4, the proposed hierarchical framework approach for VAD and speech enhancement is presented in detail. The experimental results and performance evaluation are presented in Section 5. Finally, Section 6 gives a summary of the paper.

## 2. Speech Enhancement

### 2.1. Modified Wiener Filter (MWF)

The well-known noise reduction algorithms, such as Wiener filter (WF) algorithms, are widely used for robust voice activity detection which is the critical step for speech recognition. Currently, most of the WF-based algorithms are iterative since the estimation of power spectrum of the clean speech is required in the formulation. It suffers rapid performance degradation when the driving conditions get noisier. Recently, a noniterative WF-based algorithm called modified Wiener filtering algorithm was proposed by Arslan [16]. The main advantage of the proposed method is that it makes use of a time-varying signal-to-noise ratio dependent noise suppression factor. This property increases the ability to suppress the parts of the degraded signal, where speech is not likely to be present and not to suppress, and hence not to distort the speech segments as much. The basic idea behind modified Wiener filtering is to emphasize the frequencies where speech signal is dominant over the noise signal and to attenuate the frequencies where the speech signal is weak relative to noise. The followed section presents a basic explanation of the MWF algorithm.

Suppose that the noise is additive; the noisy signal could be expressed as

(1) $$\begin{array}{c} y\left(n\right)=x\left(n\right)+d\left(n\right), \end{array}$$

where *y*(*n*) is noisy speech, *x*(*n*) is noise-free speech, and *d*(*n*) is additive noise signal. Then, a generalized Wiener filter can be formulated as

(2) $$\begin{array}{c} H\left(\omega \right)={\left(\frac{{\hat{P}}_{x}\left(\omega \right)}{{\hat{P}}_{x}\left(\omega \right)+\alpha P_{d}\left(\omega \right)}\right)}^{\beta }, \end{array}$$

where ${\hat{P}}_{x}(\omega )$ is the estimation of the clean speech power spectrum and *P*_*d*_(*ω*) is the noise power spectrum. Parameters *α* and *β* are the noise reduction factor and the power of the filter, respectively (*α* = *β* = 1 in our evaluations). We assumed that the speech and the noise signal have the same phase, so the filter adjusts the amplitude at each frequency and preserves the original phase. Then, the Fourier transform (FT) was taken:

(3) $$\begin{array}{c} \hat{X}\left(\omega \right)=H\left(\omega \right)Y\left(\omega \right),\\ \hat{x}\left(t\right)=F^{-1}\left\{\hat{X}\left(\omega \right)\right\}, \end{array}$$

where $\hat{X}(\omega )$ and *Y*(*ω*) are the estimation of the FT of the clean speech and noisy speech. *F* ^−1^{·} is the inverse Fourier transform operation. In this formulation, it is assumed that we have an estimate of the clean speech power spectrum, ${\hat{P}}_{x}(\omega )$. This estimate is calculated from the AR smoothed spectrum of the noisy speech, *P*_*y*_(*ω*), by only a DC gain modification (note that ${\hat{P}}_{x}(\omega )$ uses original shape of *P*_*y*_(*ω*) as)

(4) $$\begin{array}{c} {\hat{P}}_{x}\left(\omega \right)=\frac{{\hat{g}}_{x}^{2}}{g_{y}^{2}}P_{y}\left(\omega \right), \end{array}$$

where ${\hat{g}}_{x}^{2}$ and *g*_*y*_^2^ are the DC power of the clean speech and of the noisy speech, respectively. Assume the speech and noise signal are uncorrelated; then we have

(5) $$\begin{array}{c} P_{y}\left(\omega \right)={\hat{P}}_{x}\left(\omega \right)+P_{n}\left(\omega \right). \end{array}$$

By integrating both sides of (4) and (5) over *ω*, we get

(6) $$\begin{array}{c} \overset{\pi }{\underset{-\pi }{\int }}P_{y}\left(\omega \right)d\omega =\overset{\pi }{\underset{-\pi }{\int }}\frac{{\hat{g}}_{x}^{2}}{g_{y}^{2}}P_{y}\left(\omega \right)d\omega +\overset{\pi }{\underset{-\pi }{\int }}P_{n}\left(\omega \right)d\omega . \end{array}$$

Then, the above equation can be simplified by using Parseval's relation

(7) $$\begin{array}{c} \frac{{\hat{g}}_{x}^{2}}{g_{y}^{2}}=\{\begin{array}{cc} 1-\frac{E_{n}}{E_{y}}, & \text{if}\;\;E_{y}>E_{n},\\ 0, & \text{otherwise}, \end{array} \end{array}$$

where *E*_*n*_ and *E*_*y*_ are the energy measurement of the noise-free speech and the noisy speech. By substituting the expression for ${\hat{g}}_{x}^{2}/g_{y}^{2}$ in (4), the clean speech spectrum estimate now can be expressed as

(8) $$\begin{array}{c} {\hat{P}}_{x}\left(\omega \right)=\left(1-\frac{E_{n}}{E_{y}}\right)P_{y}\left(\omega \right). \end{array}$$

With the above expression in (2) and introducing a time-dependent noise suppression factor and after simplifications, we get

(9) $$\begin{array}{c} H\left(\omega \right)=\left(\frac{P_{y}\left(\omega \right)}{P_{y}\left(\omega \right)+\left[E_{y}/\left(E_{y}-E_{n}\right)\right]P_{n}\left(\omega \right)}\right). \end{array}$$

The result *H*(*ω*) of the above equation is always nonnegative, whereas in spectral subtraction, the equivalent filter may fluctuate between negative and positive values. Although the negative values are set to zero, there are still some sharp discontinuities in frequency domain. To further reduce the discontinuities after the clamping, a five-point moving-average smoothing is done on *H*(*ω*) [16].

### 2.2. Decision Directed Approach

In this section, we will consider the problem of estimating the a priori SNR of a spectral component in a given segment. Due to the nonstationary of the speech signal, the a priori SNR should be reestimated in each speech frame. The decision directed (DD) method is a widely used approach to determine the a priori SNR from noisy speech and it is found to be very useful, while combining with the Wiener amplitude estimator [17]. So, the estimator used in this study is based on decision directed estimation method.

Let *ξ*(*m*, *k*), *A*(*m*, *k*), *λ*_*d*_(*k*, *m*), and *γ*(*m*, *k*) denote the a priori SNR, the amplitude, the noise variance, and the a posteriori SNR of the corresponding *k*th spectral component in the *m*th speech frame, respectively. The definition of *ξ*(*m*, *k*) and its relation to the *γ*(*m*, *k*) are given below:

(10) $$\begin{array}{c} \xi \left(m,k\right)=\frac{E\left\{{\left[A\left(m,k\right)\right]}^{2}\right\}}{\lambda_{d}\left(k,m\right)},\\ \xi \left(m,k\right)=E\left\{\gamma \left(m,k\right)-1\right\}. \end{array}$$

Then the a priori SNR is defined as a linear combination of (10) with a weighting parameter *α*, which is constrained in the interval of (0,1):

(11) $$\begin{array}{c} \xi \left(m,k\right)=E\left\{\alpha \frac{{\left[A\left(m,k\right)\right]}^{2}}{\lambda_{d}\left(k,m\right)}+\left(1-\alpha \right)\left[\gamma \left(m,k\right)-1\right]\right\}. \end{array}$$

To implement efficiency in practice, an approximation was made as given below:

(12) $$\begin{array}{c} \hat{\xi }\left(m,k\right)=\alpha \frac{{\left[\hat{A}\left(m-1,k\right)\right]}^{2}}{\lambda_{d}\left(k,m-1\right)}+\left(1-\alpha \right)P\left[\gamma \left(m,k\right)-1\right], \end{array}$$

where *P*[·] is an operator which is defined by

(13) $$\begin{array}{c} P\left[x\right]=\{\begin{array}{cc} x, & \text{if}\;\;x\geq 0,\\ 0, & \text{otherwise}. \end{array} \end{array}$$

The important characteristic of the DD approach is the dependency on previously enhanced frames which results in biased estimates of the a priori SNR during speech transitions. This method results in significant elimination of musical noise [18].

## 3. Enhanced Voice Activity Detection Algorithm

In general, voice activity detectors extract certain features from the input signal for classification. These features maybe have good traits in the system but cannot meet performance expectations independently. So feature combination becomes one of the classical approaches in such systems. Since the inclusion of different features brings out additional computation cost, the feature selection process should be conducted very carefully in VAD algorithm. Considering the computational complexity and the discriminative contribution, three types of measure were selected in this study. The feature selection process is based on the features' performance for VAD systems under various noise types. They are the energy measure, the spectral entropy, and the harmonic structure-related features.

### 3.1. Energy-Based VAD

The energy-based VAD techniques are the most popular techniques and are widely used in speech recognition application. A variety of energy-based algorithms have been proposed for detecting robust voice activity in the last decades [3, 5]. With very little computational complexity, these techniques have good performance for clean speeches or speeches with little noise contamination. The implementation steps of the energy-based VAD algorithms are given as follows.

First, an audio stream was divided into nonoverlapping small segments by using a rectangular moving window. The sampling frequency was usually set as 8000 Hz. These small segments are so-called audio segments. And then, the energy level of each audio segment is calculated as follows:

(14) $$\begin{array}{c} E_{s}=\sqrt{\overset{N-1}{\underset{n=0}{\sum }}{\left[x\left(n\right)\right]}^{2}}, \end{array}$$

where *N* is the size of the audio segment.

After the energy of all the speech frames was computed, an empirical predefined threshold th is selected from the frame energies. Finally, audio segments whose energy is larger than th are sent to the feature extraction step for classification and the others are dropped for saving processing resources. Energy-based VAD techniques are very straightforward and somewhat suitable for clean conditions. The performance of the system degrades significantly under low SNR conditions when used independently.

### 3.2. Spectral Entropy

The spectral entropy is a measure of uncertainty for intrinsic characteristics of speech spectrums. Many existed experiments have proved that spectral entropy is superior to the other features under low SNR conditions, such as short-term energy and zero crossing rate [4, 14]. In the case of speech signals, the energy of certain phonemes is concentrated in a handful of spectral bands. The entropy will be low when the signal spectrum is more organized during speech segments, while it will be higher in the case of noise.

The implementation for calculating spectral entropy parameters is described as follows. First, speech signals are segmented into frames and prefiltered by modified Wiener filter as mentioned above. Each frame is evenly divided into four subframes. For each subband, the short-time Fourier transform of a given speech frame is defined as

(15) $$\begin{array}{c} X\left(k\right)=\overset{N}{\underset{n=1}{\sum }}x\left(n\right)\exp\left(\frac{-j2\pi nk}{N}\right),\;k=1,2,\ldots ,N, \end{array}$$

where *X*(*k*) represents the spectral magnitude of the *k*th frequency bin and *N* is the total number of frequency bins for each frequency frame (*N* = 128 was used in the proposed approach).

The spectral energy of each frame, *E*(*k*), is calculated by

(16) $$\begin{array}{c} E\left(k\right)={\left|X\left(k\right)\right|}^{2},\;k=1,2,\ldots ,\frac{N}{2}. \end{array}$$

And the probability measure in the spectral domain can be written by the following formula:

(17) $$\begin{array}{c} P\left(i\right)=\frac{E\left(i\right)}{\sum_{k=1}^{N/2}E\left(k\right)},\;i=1,2,\ldots ,\frac{N}{2}. \end{array}$$

Then, the corresponding spectral entropy for each subframe speech signal is defined as follows:

(18) $$\begin{array}{c} H=-\overset{N/2}{\underset{i=1}{\sum }}p\left(i\right)\cdot \log\left[p\left(i\right)\right]. \end{array}$$

Finally, the spectral entropy for each frame can be calculated as the average of four subframes. The foregoing calculation of the spectral entropy parameter implies that the spectral entropy depends only on the variation of the spectral energy but not on the amount of spectral energy. Consequently, the spectral entropy parameter is robust against changing level of noise [19].

### 3.3. Harmonic Structure Information

Mel-frequency cepstral coefficient (MFCC) features were one of the most popular used features in speech/speaker recognition applications. Mel scale takes the mechanism of the auditory nonlinear frequency resolution into consideration, which was believed to provide pertinent cues for phonetic classification in speech recognition. However, static features such as MFCC do not have sufficient information to distinguish speech from noise frames because a static MFCC sequence often has similar feature vectors for speech and nonspeech [7]. Recently, many advanced techniques were proposed to eliminate the limitation of MFCC. The harmonic structure-related information is robust to high-pitched sounds and seems to be a promising cue to increase robustness of the noise, especially in low SNR conditions [13]. Fukuda et al. [8] replaced the traditional Mel-frequency cepstral coefficients by the harmonic structure information and made a significant improvement of recognition rate. Thus, in this paper, features extracted from the harmonic structure information are introduced as an alternative to MFCC. The outline of the harmonic structure-related features is shown as follows.

First, calculate the power spectrum *x*_*t*_(*j*) and convert it into log power spectrum *y*_*t*_(*j*) for each speech segment. And after that, convert *y*_*t*_(*j*) into the cepstrum *p*_*t*_(*j*) by using the discrete cosine transform (DCT):

(19) $$\begin{array}{c} p_{t}\left(i\right)=\sum_{i}M_{a}\left(i,j\right)\cdot y_{t}\left(j\right), \end{array}$$

where *M*_*a*_(*i*, *j*) is the matrix of DCT, *i* and *j* indicate the bin index of the cepstral coefficients, and *t* is the frame number.

Then, the harmonic structure information *q*_*t*_(*i*) is obtained by lifting out the lower and higher cepstra because the medium cepstra capture the harmonic structure information:

(20) qt(i)=λ·pt(i) if  (DL<i<DH)qt(i)=pt(i) otherwise,

where *λ* is a small constant. *D*_*L*_ and *D*_*H*_ correspond to the bin index of the *F*_0_ range.

After lifting out the lower and upper cepstra, *q*_*t*_(*i*) is converted back to log power spectrum domain ${\hat{\omega }}_{t}(j)$ by inverse DCT (IDCT) and exponential transform:

(21) $$\begin{array}{c} {\hat{\omega }}_{t}\left(j\right)=\exp\left\{\sum_{i}M_{a}^{-1}\left(j,i\right)\cdot q_{t}\left(j\right)\right\}. \end{array}$$

Finally, convert *b*_*t*_(*k*) into the harmonic structure based coefficients *c*_*t*_(*n*) by using the *N* × *K* size DCT matrix *b*_*t*_(*k*):

(22) $$\begin{array}{c} c_{t}\left(n\right)=\overset{K}{\underset{k=1}{\sum }}M_{b}\left(n,k\right)\cdot b_{t}\left(k\right). \end{array}$$

### 3.4. Classification

Support vector machine (SVM) is an effective binary classification technique on the foundation of statistical learning theory [20]. In the SVM method, a unique principal called structural risk minimization (SRM) principle is used to minimize an upper bound of the generalization error. For the nonlinear case, SVM maps the training data into a higher dimensional feature space (maybe infinite) to make the input data linearly separable. Hence, we can find the hyperplane that optimally separates the dataset in the higher dimensional feature space. According to functional theory, three types of kernel functions, named polynomial kernel function, radial basis function (RBF), and sigmoid function, are usually used. In our experiment, we chose RBF as kernel functions because it always yields better classification results. The formula for computing RBF is listed below:

(23) $$\begin{array}{c} K\left(a,b\right)=\exp\left(-\gamma {||a-b||}^{2}\right). \end{array}$$

## 4. Proposed Hierarchical Framework

### 4.1. Framework Overview

**Figure 1:**
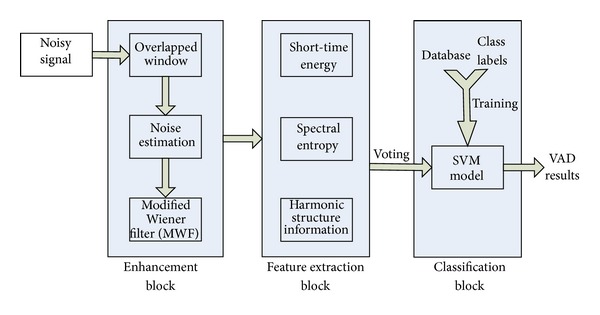
Hierarchical framework of proposed system.

It is well-known that different methods have their advantages and disadvantages under different noises conditions. In order to combine the advantages together, we proposed a hierarchical framework approach to achieve robust voice activity detection and speech enhancement. The proposed system was composed of three blocks (see Figure 1). In the speech enhancement block, a priori SNR is estimated by the decision directed method and the modified Wiener filtering approach is utilized for noise reduction. For the feature extraction and voting block, several discriminating short-term features were extracted in various noisy conditions. Since some features are more appropriate for a specific noisy condition, these features were then employed in a voting paradigm for the consideration of reliability and discriminative power. Two speech corpuses are employed for training the SVM models under different SNR levels in the training/classification block. Finally, the “one-against-one” approach of support vector machine was utilized for separating speech/nonspeech segments.

### 4.2. Voting Paradigm

Since each feature has its relative contribution in the decision process and the performance may be different under different noisy conditions, the feature voting scheme seems to be the inevitable way to improve the degree of reliability and discriminative power. Voting approach can satisfy the performance expectations and the computational complexity with a certain degree of robustness. Recent studies have shown that voting approach leads to more robust results than the simple combination ones. In this paper, selected features were employed in a voting paradigm for the consideration of reliability and discriminative power. For each signal segment, the three features are computed. If more than one of the feature values exceed the given threshold, the segment is marked as a speech frame. Experiment results were compared to the baseline approaches under various noisy conditions.

## 5. Experiment and Results

### 5.1. Database Description

In this section, the performance of the proposed voice activity detection and speech enhancement algorithm is evaluated on the experiment data. The experiment data come from two well-known databases, TIMIT database and NOISEX-92 database. TIMIT corpus consists of 1680 utterances from 168 individual speakers. A subset of the TIMIT database, 90 short sentences from six speakers, is used for our experiments. Four typical types of noises, namely, white noise, buccaneer noise (F16 cockpit), babble noises, and factory noise, from the NOISEX-92 database are artificially added to the test dataset at different SNR conditions.

### 5.2. Performance Evaluation

In order to evaluate the performance of the proposed VAD algorithm, experiment results were analyzed using two common metrics which are known as nonspeech (silence) hit rate (HR0) and speech hit rate (HR1). They are defined as the ratio of the detected nonspeech (silence) or speech frames to the total number of nonspeech (silence) or speech frames, respectively. The two metrics were presented as a function of the SNR to clearly describe the performance of the proposed VAD algorithm. For the analysis, the actual number of speech and nonspeech frames was determined by the ideal VAD:

(24) HR0=number  of  nonspeech  frames  correctly  classifiednumber  of  real  nonspeech  frames ×100%,HR1=number  of  speech  frames  correctly  classifiednumber  of  real  speech  frames ×100%.

Since there are always trade-off relationships among these two metrics, we use the mean of HR0 and HR1 as the final metric for better performance comparison.

### 5.3. Implementation

In our implementation, the input speech signals were divided into nonoverlapping frame segments of unit length by using rectangular window of fixed height. For the input speech/noise signals sampled at 8000 Hz, the segments are of 32 ms each. Frame segments were marked as silent frames or speech frames by using a predefined empirical threshold. Audio segments whose energy is larger than th are sent to the feature extraction step for classification, and silent frame segments are automatically removed.

**Figure 2:**
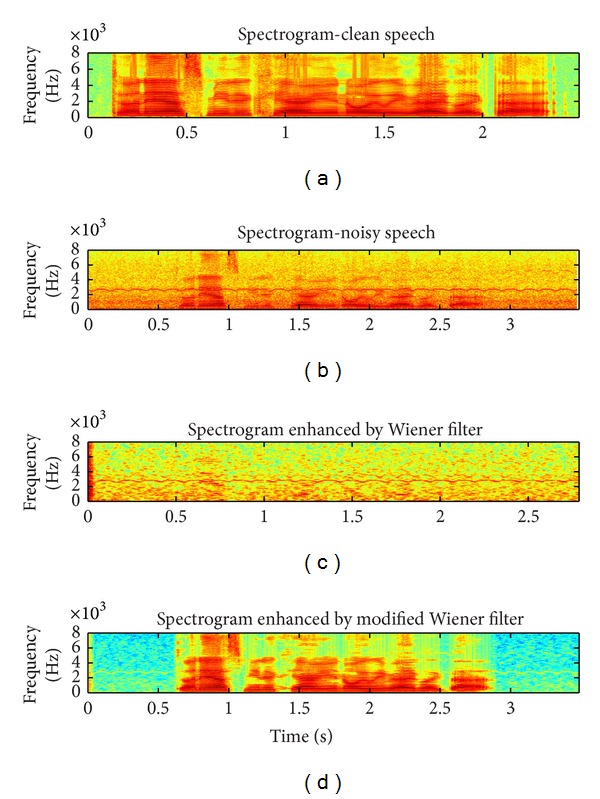
Speech spectrograms, buccaneer noise, 10 dB. From (a) to (d), clean signal, noisy signal, enhanced signal by Wiener filter, and enhanced signal by modified Wiener filter.

Figure 2 displays the spectrograms of clean speech, noisy speech, result by using Wiener filter, and result by modified Wiener filter, respectively. The speech is “Do not ask me to carry an oily rag like that” and it was corrupted by buccaneer noise at 10 dB. It is observed from Figure 2 that musical residual noise is almost all removed in most parts of the noisy speech.

**Figure 3:**
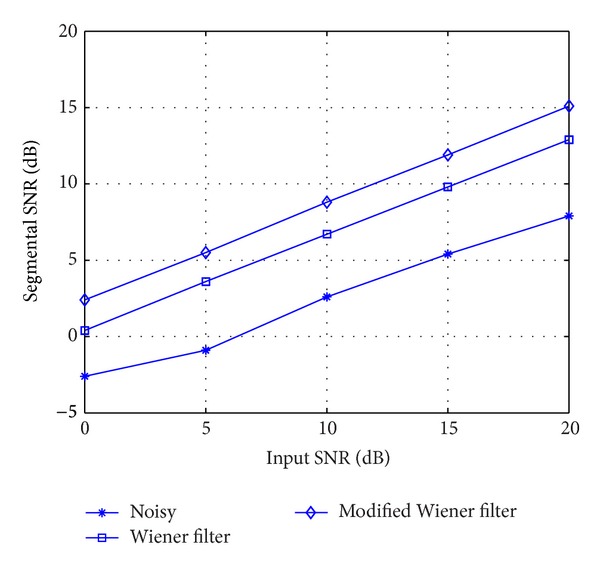
Results of segmental SNR measures of noisy signal, the Wiener filter, and the modified Wiener filter.

Figure 3 shows the input and output segmental SNR of noisy signal, the Wiener filter, and the modified Wiener filter. The segmental SNR is one of the objective quality measures used for the evaluation of the speech enhancement algorithms. It is more accurate in indicating the speech distortion than the overall SNR. For the segmental SNR, higher value always indicates less speech distortions. The degraded speech signal was added by white noise from the NOISEX-92 database. It is observed in Figure 3 that the MWF yields better SNR than that of the Wiener filtering technique under noisy environments.

**Figure 4:**
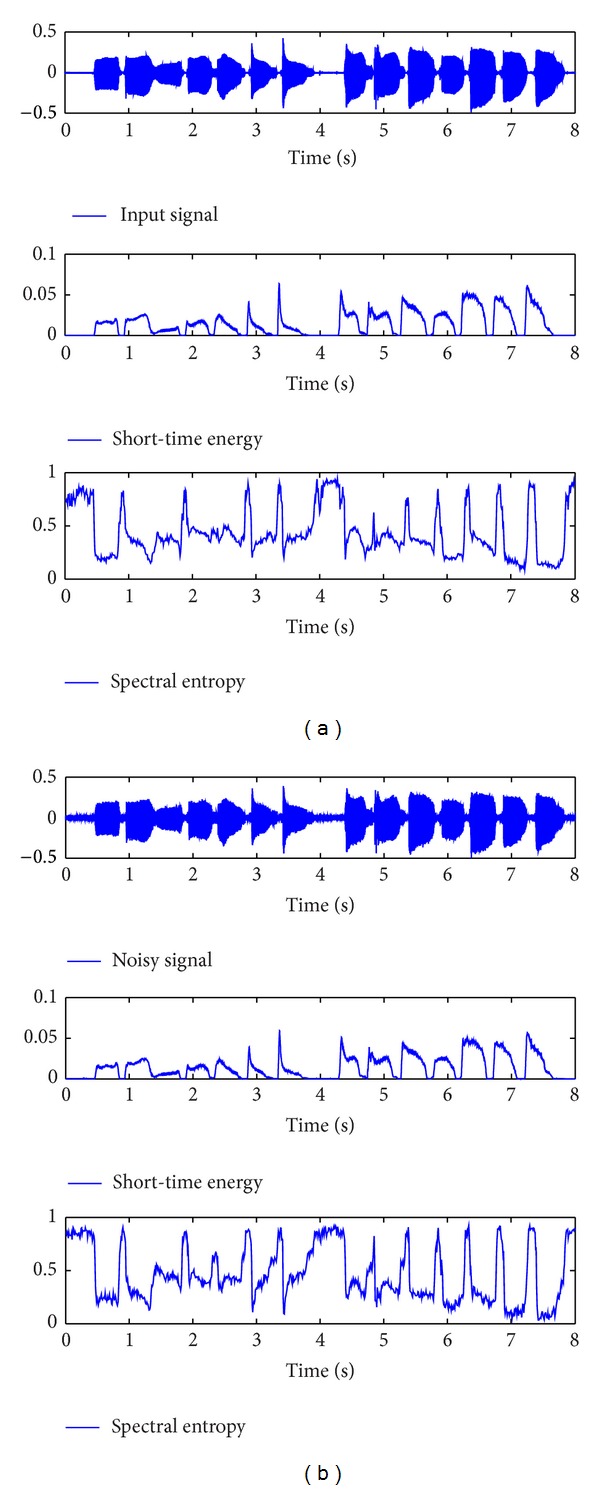
Clean/noisy signal and its correspondence short-term energy and spectral entropy.

Figure 4(a) shows a clean speech signal and its correspondent results by using STE (short-time energy) and SE (spectral entropy). The test speech signal contains 250 frames at the sampling frequency of 8 kHz and is segmented into small segments with nonoverlap.  Figure 4(b) shows a noisy speech signal and its correspondent results by using STE and SE. The noisy speech signal was obtained from the clean speech signal by adding buccaneer noise at SNR = 10 dB.

In this study, we perform LIBSVM for the classification step. The LIBSVM is a library for support vector machines which is available in [21]. For the implementation of SVM, the radial basis function (RBF) kernel was used to map nonlinearly data into a higher dimensional feature space. By using the LIBSVM, one-against-one algorithm was performed to find the best parameters of the support vectors, and then the parameters were used to train the whole training set. Finally, a cross-validation method was utilized to analyze the classification performance.

To compare the performance for all kinds of conditions, we introduced the average speech/nonspeech hit rates under different noise conditions at 5 dB.  Table 1 shows the results obtained with the G.729 [22], AMR2 [23], and proposed approach. These results are then averaged in the last column. In Table 1, we observe that the average accuracy under various noises of the proposed VAD is always the best among all reference VAD algorithms, especially in white noise. The proposed approach achieved better performance in detecting speech with a 75.3% average value.

Table 2 shows the performance of proposed VAD algorithm at various SNR levels. Experimental results show that the proposed algorithm shows stable performance across various noisy conditions. The average results of different SNR levels are listed in the last line.

From Table 2, we can see that accuracy of the proposed VAD algorithm increased from 66.8% to 97.8% when SNR levels increased from 0 dB to 20 dB. As can be seen from the results, the proposed algorithm based on modified Wiener filter and the voting paradigm achieved a good performance, even at low SNR levels.

## 6. Conclusion

In this study, we proposed a hierarchical framework approach for robust VAD and speech enhancement. The proposed system composed of three blocks, namely, the speech enhancement block, the feature extraction and voting block, and the training/classification block. Modified Wiener filter approach is utilized for noise reduction and has been shown to perform better than ordinary Wiener filter in all tested noisy conditions. And then, several discriminate features and a well-trained SVM were employed in a voting paradigm to identify the speech or nonspeech segments. Finally, the proposed method was objectively evaluated in four kinds of noises at various SNR levels. The proposed approach is also compared with some other VAD techniques for better confirmation of its achievements. Experimental results show that the proposed algorithm performs well under a variety of noisy conditions.

## Figures and Tables

**Table 1:** Average speech/nonspeech hit rates under different noise conditions at 5 dB.

VAD	Noise				
	White	Buccaneer	Babble	Factory	Average
G.729	80.4%	49.4%	51.6%	57.1%	59.6%
Amr2	75.5%	63.3%	64.2%	64.5%	66.9%
Proposed	87.9%	69.7%	70.3%	73.2%	75.3%

**Table 2:** Accuracy of the proposed VAD algorithm at different SNR levels.

Noise	SNR				
	0 dB	5 dB	10 dB	15 dB	20 dB
White noise	53.3%	61.7%	67.1%	94.5%	97.8%
Buccaneer noise	67.6%	75.4%	84.1%	90.8%	96.5%
Babble noises	84.2%	88.3%	93.2%	94.7%	99.1%
Factory noise	61.9%	69.5%	82.9%	97.5%	98.0%

Average	66.8%	73.7%	81.8%	94.4%	97.8%
